# Functional Effects of Different Medium-Chain Acyl-CoA Dehydrogenase Genotypes and Identification of Asymptomatic Variants

**DOI:** 10.1371/journal.pone.0045110

**Published:** 2012-09-17

**Authors:** Marga Sturm, Diran Herebian, Martina Mueller, Maurice D. Laryea, Ute Spiekerkoetter

**Affiliations:** Department of General Pediatrics, University Childreńs Hospital, Düsseldorf, Germany; University of Louisville, United States of America

## Abstract

Medium-chain acyl-CoA dehydrogenase (MCAD) deficiency (OMIM 201450) is the most common inherited disorder of fatty acid metabolism presenting with hypoglycaemia, hepatopathy and Reye-like symptoms during catabolism. In the past, the majority of patients carried the prevalent c.985A>G mutation in the *ACADM* gene. Since the introduction of newborn screening many other mutations with unknown clinical relevance have been identified in asymptomatic newborns. In order to identify functional effects of these mutant genotypes we correlated residual MCAD (OMIM 607008) activities as measured by octanoyl-CoA oxidation in lymphocytes with both genotype and relevant medical reports in 65 newborns harbouring mutant alleles. We identified true disease-causing mutations with residual activities of 0 to 20%. In individuals carrying the c.199T>C or c.127G>A mutation on one allele, residual activities were much higher and in the range of heterozygotes (31%–60%). Therefore, both mutations cannot clearly be associated with a clinical phenotype. This demonstrates a correlation between the octanoyl-CoA oxidation rate in lymphocytes and the clinical outcome. With newborn screening, the natural course of disease is difficult to assess. The octanoyl-CoA oxidation rate, therefore, allows a risk assessment at birth and the identification of new *ACADM* genotypes associated with asymptomatic disease variants.

## Introduction

Medium-chain acyl-CoA dehydrogenase (MCAD) deficiency (OMIM 201450), initially identified in 1982 [1], is the most common autosomal recessive disorder of the mitochondrial ß-oxidation with a regional incidence of 1∶9,000 to 1∶10,000 in Northern Europe [2] and has been part of many newborn screening programs for the past ten years [3]–[5]. MCAD (EC 1.3.99.3) catalyzes the oxidation of fatty acids with chain lengths between C4 and C12 [6]–[8]. The clinical phenotype is generally induced by catabolism. Increased energy demand beyond the availability of glucose, prolonged fasting, illness or childhood immunization may induce metabolic derangement with clinical symptoms such as hypoketotic hypoglycemia, hepatic dysfunction and neurological impairment [3], [5], [9]–[12]. Severe metabolic crisis may result in hepatic failure and Reye-like features leading to coma and death [3], [9], [10], [13]. Newborns with elevated octanoyl-carnitine levels in the newborn screen go generally for genetic tests and additionally enzymatic activity measurements (AWMF guidelines DGKJ, No. 027/021, http://www.awmf.org). Before newborn screening when MCAD was identified on the basis of clinical findings, c.985A>G was identified as the most prevalent mutation in the *ACADM* gene (NM_000016.4) where approximately 80% of patients exhibited homozygosity for this change. With the advent of newborn screening, where many newborns are identified in the absence of a clinical presentation, a second prevalent mutation was identified c.199T>C (allele frequency approximately 6%). This mutation however, has never been conclusively linked to clinical symptoms [7], [9], [10], [14], [15]. Instead, most children with the c.199T>C allele are placed on preventative measures, which potentially masks the natural clinical course of the disease. Further, this mutation has only been identified in the heterozygous state raising questions as to whether it may even be responsible for the elevated octanoyl-carnitines identified in the newborn screen.

As in other metabolic enzyme deficiencies with implementation of newborn screening many asymptomatic “patients” are reported. Therefore it would be of great relevance to identify parameters which could serve as reliable predictors of disease severity. The distinction between patients at risk of symptomatic disease and individuals who may never develop symptomatic disease in the long term is very important. This distinction is especially important for novel mutations where prophylactic measures may be implemented without having an adequate assessment that the mutation will lead to clinically significant disease presentation.

Here we analyzed the genetic basis for the increase in octanoyl-carnitine in 65 individuals analyzed by newborn screening. This analysis included activity measurements of the MCAD enzyme in lymphocytes. We correlated the measured MCAD activity with both mutant genotypes and natural clinical presentation described in relevant medical reports.

**Table 1:** Genotype and octanoyl-CoA oxidation measured in lymphocytes of 65 subjects with suspected MCAD deficiency^a^.

patient	allele 1	allele 2	HPLC-UV [%]	HPLC-ESI-MS/MS [%]	C8∶0-carnitine [µmol/L]^b^
1	WT	WT	57	89	–c
2	WT	WT	72	102	–c
3	WT	WT	79	99	–c
4	WT	WT	99	114	–c
5	c.985A>G	WT	50	56	–c
6	c.985A>G	WT	57	56	–c
7	c.985A>G	WT	60	60	–c
8	c.985A>G	WT	75	87	–c
9	c.985A>G	WT	32	55	–c
10	c.985A>G	WT	20	33	–c
11	c.985A>G	WT	12	22	–c
12	c.985A>G	WT	13	24	0.36 (<0.3)
13	c.199T>C	WT	99	114	–c
14	c.199T>C	WT	–d	80	–c
15	c.245–246insT	WT	40	53	–c
16	c.737delA	WT	28	52	0.58 (<0.18)
17	c.985A>G	c.985A>G	0	2	–c
18	c.985A>G	c.985A>G	0	2	–c
19	c.985A>G	c.985A>G	0	0	3.35 (<0.13)
20	c.985A>G	c.985A>G	0	4	7.12 (<0.3)
21	c.985A>G	c.985A>G	0	4	–c
22	c.985A>G	c.985A>G	0	4	19.2 (<0.3)
23	c.985A>G	c.985A>G	0	4	–c
24	c.985A>G	c.985A>G	0	5	48.6 (<0.3)
25	c.985A>G	c.985A>G	0	5	7.2 (<0.41)
26	c.985A>G	c.985A>G	0	5	2.91 (<0.3)
27	c.985A>G	c.985A>G	0	3	0.88 (<0.3)
28	c.985A>G	c.985A>G	0	7	4.6 (<0.3)
29	c.985A>G	c.985A>G	0	8	4.99 (<0.34)
30	c.985A>G	c.985A>G	1	6	–c
31	c.985A>G	c.985A>G	1	8	–c
32	c.985A>G	c.985A>G	2	5	–c
33	c.985A>G	c.985A>G	2	5	–c
34	c.985A>G	c.985A>G	2	5	12.0 (<0.3)
35	c.985A>G	c.985A>G	3	4	–c
36	c.985A>G	c.985A>G	3	8	13.7(<0.3)
37	c.985A>G	c.985A>G	5	5	–c
38	85C>T	85C>T	3	6	8.04 (<0.29)
39	c.245–246insT	c.245–246insT	0	4	6.01 (<0.4)
40	c.245–246insT	c.245–246insT	0	4	8.54 (<0.34)
41	c.397–399delATT	c.397–399delATT	0	3	7.88 (<0.4)
42	c.799G>A	c.799G>A	5	16	–c
43	c.799G>A	c.799G>A	0	10	5.40 (<0.3)
44	**c.1010A>C** e	**c.1010A>C** e	3	7	2.7 (<0.5)
45	**c.1010A>C** e	**c.1010A>C** e	4	10	2.41 (<0.35)
46	c.985A>G	c.233T>C	0	0	–c
47	c.985A>G	c.245–246insT	0	4	–c
48	c.985A>G	del AAG	0	5	6.17 (<0.28)
49	c.985A>G	c.464T>C	3	3	–c
50	c.985A>G	c.616C>T	12	12	7.2 (<0.3)
51	c.985A>G	**c.1033G>T** e	0	3	5.74 (<0.4)
52	c.985A>G	**c.1225C>T** e	0	6	0.93 (<0.4)
53	c.985A>G	**c.2T>G** e	2	7	–c
54	c.985A>G	**c.982A>G** e	12	0	2.94 (<0.20)
55	c.985A>G	c.127G>A	48	60	0.56 (<0.18)
56	c.985A>G	c.199T>C	16	28	4.27 (<0.3)
57	c.985A>G	c.199T>C	18	35	1.6 (<0.28)
58	c.985A>G	c.199T>C	21	33	2.0 (<0.3)
59	c.985A>G	c.199T>C	–d	26	1.82 (<0.3)
60	c.985A>G	c.199T>C	33	49	1.62 (<0.18)
61	c.799G>A	c.199T>C	19	31	–c
62	c.799G>A	**c.533A>C** e	11	20	2.5 (<0.35)
63	c.1114–1115insG	IVS 5+2–3insG	0	4	–c
64	c.977T>C	c.698T>C	8	15	–c
65	IVS 5+1 del G	**c.1229T>G** e	0	7	10.2 (<0.3)

a Activity was given as % of the mean value of healthy controls (1.89±0.58 mU/mg).

b Newborn screening octanoyl-carnitine results were given in µmol/L. The cut-off values were given bracketed.

c Data not available.

d no HPLC-UV measurement performed.

e novel mutation.

## Methods

### Ethics Statement

The study was performed according to the rules of the Declaration of Helsinki and approved under study number 3827 by the ethical review board of Heinrich-Heine-University Duesseldorf. Informed consent from the patients or their parents was obtained by the attending physicians in oral agreement, as the determination of MCAD residual activity constitutes a routine procedure carried out by our laboratory as part of confirmation diagnostics.

### Recruiting Newborns and Controls

Sixty-five newborns with conspicuous initial blood octanoyl-carnitine concentrations on NBS and therefore suspected MCAD deficiency were analyzed. Samples were received from different pediatric hospitals or Pediatricians in Germany to confirm or invalidate MCAD deficiency by measuring residual MCAD activity in lymphocytes and performing molecular analysis of the *ACADM* gene. Control samples were collected from anonymous healthy volunteers without evidence of metabolic disease. Samples were also obtained from known heterozygous parents.

Octanoyl-CoA oxidation in lymphocytes and sequencing of the *ACADM* gene are regular confirmatory diagnostic tools in case of suspected MCAD deficiency on NBS (AWMF guidelines DGKJ, No. 027/021, http://www.awmf.org). For those patients harboring two mutations, parents were analyzed to determine whether mutant alleles were derived from a parent heterozygous for a mutant allele.

**Figure 1:**
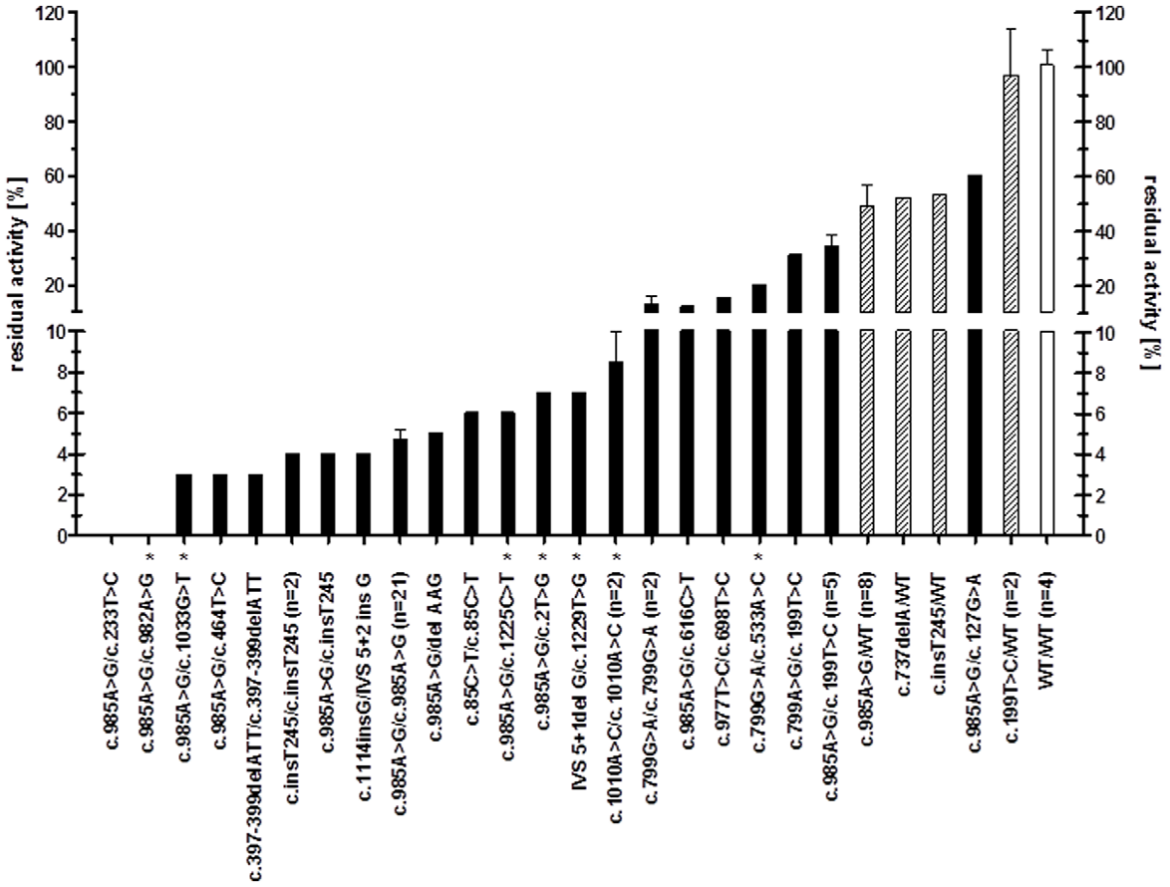
Correlation of octanoyl-CoA oxidation with *ACADM* genotype. The patients are grouped into 1. Wild-types, 2. Individuals with only one mutation found and 3. Homozygous and compound heterozygous newborns. Patients with identical genotypes are grouped in one bar. Homozygotes and compound heterozygotes are shown in black, heterozygotes in shaded and wild-types in white bars. * denotes novel identified missense mutations. Relative residual octanoyl-CoA oxidation activities are presented as a percentage of the mean value of healthy controls ± standard error of the mean (SEM).

### ACADM Gene Analysis and in silico Prediction

To determine the genotype, genomic DNA was extracted from EDTA blood with QIAmp®. DNA Mini Kit (Qiagen, Hilden, Germany). All exons and parts of the neighboring intron regions of the *ACADM* gene were amplified by polymerase chain reaction [4]. (Primer sequences are available on request.) The products were separated by agarose gel electrophoresis and purified with the QIAquick® Gel Extraction Kit (Qiagen, Hilden, Germany). In a second step, all exons were amplified using only forward or reverse primer. They were purified by DyeEx® 2.0 Spin Kit (Qiagen, Hilden, Germany) and subsequently sequenced with an ABI Prism™ 310 genetic Analyzer.

Single base pair variations to the *ACADM* gene sequence (NM_000016.4) were assessed for pathogenicity by performing different *in silico* analysis with POLYPHEN (http://genetics.bwh.harvard.edu/pph/), SNAP (http://www.rostlab.org/services/SNAP/submit), Pmut (http://mmb2.pcb.ub.es:8080/PMut/) and MUpro (http://www.ics.uci.edu/~baldig/mutation.html). All discovered mutations were compared to dates of The Human Gene Mutation Database (HGMD®; http://www.hgmd.cf.ac.uk/ac/index.php) and the corresponding literature links. Data were submitted as supplemental information in Table S1.

**Figure 2:**
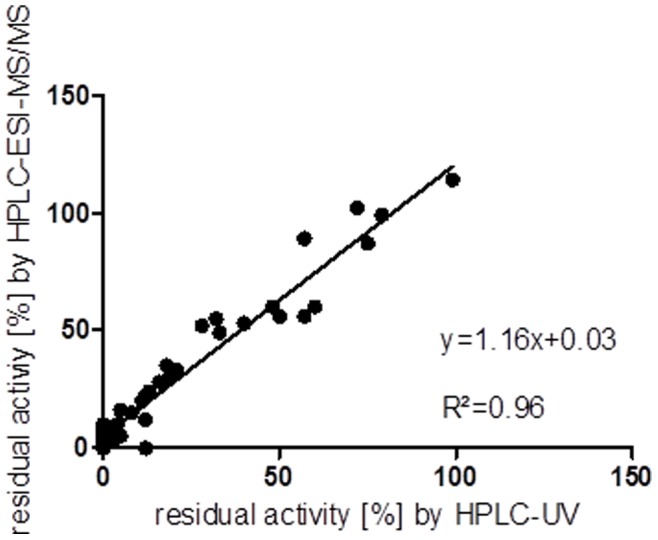
Correlation of octanoyl-CoA oxidation residual activities [%] detected by HPLC-UV and by HPLC-ESI-MS/MS. Relative residual octanoyl-CoA oxidation activities are presented as a percentage of the mean value of healthy controls. The resulting regression line is y = 1.16×+0.03. The correlation coefficient R^2^ is 0.96.

### Lymphocyte Isolation and Octanoyl-CoA Oxidation

Detection of the MCAD residual activity was achieved by assaying the oxidation of octanoyl-CoA in lymphocytes. The isolation of lymphocytes from 1 to 2 ml of EDTA blood was performed using Ficoll-PaqueTM Plus (Amersham Biosciences, Freiburg, Germany) and Leucosep H (Greiner Bio One, Solingen, Germany) tubes, as described before [16]. The isolated lymphocytes were stored at -80°C. Oxidation of octanoyl-CoA was performed as previously reported [17] with minor modifications. We started the reaction with 10 nmol octanoyl-CoA and stopped it after an incubation time of 10 min. The detection of the assay analytes were performed with two different detection techniques: high pressure liquid chromatography electrospray ionization tandem mass spectrometry (HPLC-ESI-MS/MS) and high performance liquid chromatography ultraviolet spectrophotometry detection (HPLC-UV analysis).

### HPLC-ESI-MS/MS Analysis

Injection volume of 10 µl was analyzed on a 2.1×100 mm, Luna C18 (2) 3 µm (Phenomenex) in combination with a C18 Luna security guard column at room temperature using a Waters 2795 Alliance HPLC system (Waters, Milford). An isocratic elution was performed for separation of the target analytes (C8∶0-CoA, C8∶1-CoA and C8:OH-CoA) using 60% methanol and 40% water containing 10 mM ammonium acetate and 0.1% ammonium hydroxide. The total run was 5 minutes. Following retention times were obtained: 1.8 min (C8:OH-CoA), 2.5 min (C8∶1-CoA) and 3.1 min (C8∶0-CoA). Samples were measured on the same day after the MCAD assay. The intra assay variation was less than 5%.

The tandem mass spectrometer (MS/MS) Quattro Micro (Waters Micromass technologies, Manchester, UK) was operated in the positive electrospray ionization (ESI) mode. Capillary voltage and cone voltage were set to 4000 V and 35 V, respectively. The desolvation gas was maintained at 700 L/h at a temperature of 325°C; the cone gas was maintained at 100 L/h, and the source temperature was set to 120°C. For MS/MS fragmentation experiments collision energy of 35 V was used. The target analytes were detected in the selected reaction monitoring (SRM) mode with the following mass transitions: C8:OH-CoA, m/z 910>403.2; C8∶0-CoA, m/z 894>387.2; C8∶1-CoA, m/z 892>385.2, respectively. Quantification was based on peak area ratios, with the initial substrate C8∶0-CoA amount set to 10 nmol. Raw data were processed using the software MassLynx Version 4.1 (Waters, Manchester, UK).

### HPLC-UV Analysis

The analytes C8∶0-CoA, C8∶1-CoA and C8:OH-CoA were measured based on high-performance liquid chromatography separation followed by UV-detection. For HPLC analysis a Phenomenex C18(2) Luna column (2.1×100 mm×3 µm) in combination with a C18 Luna guard column were used. The two mobile phase solvents were 25 mM KH_2_PO_4_ (pH = 6.9) and acetonitrile (MeCN). An elution gradient was used with the following settings: starting from 0 to 2 min (10% to 20% MeCN); from 2 to 3.6 min (20% to 26% MeCN); from 3.6 to 7 min (isocratically at 26% MeCN); from 7 to 9 min (26% to 10% MeCN); from 9 to 13 min (isocratically at 10% MeCN). The injection volume was 3 µl at a flow rate of 0.38 mL/min. The acyl-CoA esters were detected at 254 nm and C8∶1-CoA, C8∶0-CoA and C8:OH-CoA were eluted after 5.9, 8.1 and 8.9 min, respectively. Quantification was based on peak area ratios, with the initial substrate C8∶0-CoA amount set to 10 nmol.

### Statistical Analysis

All graphs and statistical calculations were generated using Graph Pad Prism 5.0. Data are presented as means ± standard error of the mean (SEM).

## Results

### Genotype and Effect on Enzyme Function

A total of 65 newborns were evaluated in this study. Of these, 57 presented with previously reported mutations or no mutation at all in the *ACADM* gene. Eight patients (# 44–45, 51–54, 62 and 65) had novel missense mutations in a homozygous state or in compound heterozygous state with known mutations (Table 1).

All residual activities mentioned in the text are based upon the HPLC-ESI-MS/MS measurements. HPLC-UV and HPLC-ESI-MS/MS data are listed in Table 1. This table also included initial octanoyl-carnitine levels from several newborns. Newborns 1–4 had normal residual activities (89–114%) and no mutations were found. MCAD deficiency in these cases was excluded, despite a disease-specific acylcarnitine profile on newborn screening. In 12 children (# 5–16) only one heterozygous mutation was identified despite sequencing of all exons and flanking intron regions of the *ACADM* gene. Eight of them carried the prevalent mutation c.985A>G on one allele. The mean activity of all eight children was 49%. Two children, carrying the second most prevalent mutation c.199T>C on one allele, had residual activities of 114% and 80%, respectively (# 13 and 14), and were in the range of healthy controls not carrying any *ACADM* mutation. Heterozygotes commonly presented with residual activities of >50% of normal and did not present clinical manifestations of MCAD deficiency. Therefore, clinical manifestations for those heterozygous children (# 13 and14) with residual activities higher than those of carriers are very unlikely. Two individuals (# 11 and 12) with activities of 22% and 24% may not be heterozygous and the second mutation may not have been found.

Twenty-one newborns were characterized as homozygous for the prevalent mutation c.985A>G (# 17–37). They all presented with very low residual activities ranging from 0 to 8%. Eight other patients had homozygous missense mutations or small deletions (# 38–45). Their residual activities ranged from 3% to 16%. The remaining 20 children (# 46–65) carried two different mutations one on each copy of the *ACADM* gene. Several of these children were compound heterozygotes containing one copy of the prevalent mutation c.985A>G together with known or novel missense mutations, small deletions, or insertions and had residual activities between 0–12% (# 46–54). Other children were compound heterozygotes with mutations predicted to interfere with the expression of the *ACADM* gene. These included patient 63 with one exon insertion and a splice site mutation, patients 39 and 40 with homozygous c.245–246insT insertions, and patient 65 with a small splice site deletion. Each of these patients had less than 10% residual activity.

Patients with classical *ACADM* mutations, activities ≤20% were found. In contrast, some of the patients had residual activities of >25% of normal. This group included children # 55–61 who had either c.199T>C or c.127G>A on one allele. Similarly, the genotype of # 61 (c.799G>A/c.199T>C) was associated with a relatively high residual activity of 31%. The mutation c.799G>A was also found in a second patient, # 62, this time in combination with c.533A>C giving a residual activity of 20%. Thus, the mutation c.533A>C may have a more significant effect on residual enzyme activity than mutation c.199T>C.

In conclusion, deletions and insertions resulted in significantly reduced residual activities of <10% just as in patients with homozygosity for the prevalent c.985A>G mutation. In general, other homozygous or compound heterozygous missense mutations mainly caused equivalent low residual activities ≤20%, but there were exceptions such as the mutations c.199T>C and c.127G>A (# 55–61) with much higher activities.

Identical genotypes revealed similar residual activities as displayed in Fig. 1. However, the different mutations showed a very broad range of corresponding residual activities in the heterozygous state. While four individuals without a detectable mutation presented residual activities about 100%, the group of newborns with only one identified mutation was most heterogeneous with average residual activities ranging from 49% in individuals with the prevalent mutation c.985A>G to 97% carrying the second common mutation c.199T>C on one allele. Heterozygous parents were also tested via enzyme assay and gene analysis. They revealed only one mutation and had residual activities in the same range as newborns with one mutation. These parents never developed clinical symptoms in the course of their life not even during physical exercise or illness (data not shown).

Through the course of these studies we identified seven novel missense mutations. All novel *ACADM* mutations were identified in patients with two mutations (# 44–45, 51–54, 62 and 65). Four of the newly identified missense mutations were found in combination with the prevalent severe mutation c.985A>G (# 51–54). In these patients very low residual activities below 8% occurred. The mutation c.1010A>C was found in homozygous form in two closely related children (# 44 and 45). The average residual activity was 8.5%. The novel mutation c.533A>C in combination with the c.799G>A mutation in patient 62 resulted in a fairly high residual activity of 20%, as mentioned above. The novel missense mutation c.1229T>G in combination with the new single base pair deletion IVS5-1delG showed a residual activity of 7% (# 65). In conclusion, the seven newly identified missense mutations resulted in a significant reduction of residual activities in all eight patients. These findings clearly suggest that these mutations may place a child at an increased risk for clinical manifestations of MCADD. In addition to that, eight of the previously reported missense or nonsense mutations (c.985A>G, c.85C>T, c.799G>A, c.233T>C, c.464T>C, c.616C>T, c.977T>C, c.698T>C) caused clinical symptoms in the past.

Importantly, the mutations c.199T>C and c.127G>A have never been reported to cause any clinical phenotype. In individuals compound heterozygous for one of these two mutations and another mutation residual activities in the range of 31%–60% were found. In contrast, compound heterozygotes with mutations linked to disease presentation based on literature accounts had residual activities <20% (Fig. 1). These data strongly suggest that c.199T>C and c.127G>A may be innocuous polymorphisms.

### Initial Octanoyl-carnitine Values on Newborn Screening

In order to test if the octanoyl-carnitine concentration measured on newborn screening correlates with disease, we collected relevant screening data on several newborns. Due to the fact, that different German screening laboratories with individual cut-off levels measured the initial octanoyl-carnitine levels, a comparison of these data is difficult. However, some conclusions could be drawn. In our cohort of homozygous patients for the prevalent mutation c.985A>G we received 11 octanoyl-carnitine values in a very broad range of 0.88 µmol/L (cut-off <0.3 µmol/L) (# 27) to 48.6 µmol/L (cut-off <0.3 µmol/L) (# 24). In addition, patients compound heterozygous for c.985A>G and c.199T>C revealed octanoyl -carnitine concentrations from 1.6 µmol/L (cut-off <0.28 µmol/L) (# 57) to 4.27 µmol/L (cut-off <0.3 µmol/L) (# 56). Whereas in patients with the genotype c.985A>G/c.985A>G, which leads to severe clinical presentation, octanoyl-carnitine can be highly elevated, this was not the case for the c.985A>G/c.199T>C genotype, which did not cause clinical symptoms in the past. However, since there were overlapping octanoyl-carnitine concentrations in the two groups, neither a distinct correlation between genotype and octanoyl-carnitine value nor between residual activity and octanoyl-carnitine value is given. Therefore, a mild elevation on newborn screening does not allow speculating on the expected genotype or the clinical presentation of the disease.

### Correlation of HPLC-UV and HPLC-ESI-MS/MS Analyses

To determine which technique is more adequate for the detection of the end products obtained by oxidation of octanoyl-CoA, the samples were tested with both techniques: HPLC-UV and HPLC-ESI-MS/MS. Both detection techniques led to comparable values, resulting in a regression line of y = 1.16×+0.03 and a coefficient of correlation of R^2^ = 0.96. Therefore, a correlation between both techniques is given (Fig. 2). Both methods can be used for detection of octanoyl-CoA and its resulting products.

## Discussion

In this study, we are able to clearly determine the functional impairment of the MCAD enzyme in different genotypes by measurement of octanoyl-CoA oxidation in lymphocytes. Using this technique we demonstrate that in addition to identifying children that may develop clinical features of MCAD deficiency, newborn screening coupled with genetic analysis alone may also identify children who are likely to be asymptomatic and will never go on to manifest clinically significant MCAD deficiency. Two *ACADM* variants fall in this category. Residual activity in children with these genotypes falls within the range of activities measured for heterozygote carriers of other alleles who do not carry a risk for metabolic decompensation during catabolism. The correlation of genotype and residual activity is of great clinical relevance since it allows a prediction of the expected risk of metabolic decompensation that is otherwise difficult to determine. Clinical long-term follow-up studies to answer the question of clinical relevance of mutations are difficult to undertake since all individuals with suspected MCAD deficiency identified by newborn screening receive prophylactic measures in situations of illness.

Currently, *ACADM* gene analysis is the most important tool for confirmation diagnosis in MCAD deficiency (AWMF guidelines DGKJ, No. 027/021, http://www.awmf.org). Our results, however, indicate that certain *ACADM* mutations do not categorically cause symptomatic MCAD deficiency. Residual activities in lymphocytes of compound heterozygous children carrying c.199T>C or c.127G>A on one allele were 31% and 60%. These activities are clearly in the range of proven heterozygotes that do not have a risk of symptomatic disease, unless in a situation of possible synergistic heterozygosity [18]. However, this is a different condition and does not apply here. In addition, two heterozygous newborns (# 13–14) with the genotype c.199T>C/WT presented a mean residual activity of 97%, similar to healthy controls.

Furthermore, our literature research disclosed that both mutations, c.199T>C and c.127G>A, never caused any clinical symptoms [13], [19]_._ Before the screening era these mutations have never been delineated in clinically affected patients. All these data support that both mutations, c.199T>C and c.127G>A, do not have a significant effect on MCAD function and must be classified as variants with no clinical relevance. Unfortunately, long-term clinical studies are not able to confirm this, unless prophylactic measures during illness are abandoned. From personal observati**o**n (U. Spiekerkoetter), an asymptomatic father of an MCAD-deficient child, homozygous for the c.985A>G mutation, carried the c.127G>A mutation on one allele and the prevalent c.985A>G mutation on the other. He had never been symptomatic despite many infectious illnesses in the past.

Our data further demonstrate that the octanoyl-carnitine concentration measured on newborn screening is not significant. The lack of correlation between the octanoyl-carnitine concentration on screening and genotype or residual activity in our cohort was obvious and is perfectly in line with previous reports [15], [17]. The severity of novel genotypes can neither be estimated/predicted via *ACADM* gene analysis nor via octanoyl-carnitine concentration on newborn screening. We want to highlight that the assessment of enzyme function in patients with new genotypes is the only possibility to discover whether they carry the risk for the development of a clinical phenotype or not.

In our cohort of patients seven novel missense mutations were identified. Due to the fact that all novel missense mutations were either found in homozygous state, in compound heterozygous state with the prevalent mutation c.985A>G or with another clearly pathogenic mutation, a general classification of their functional effects was possible. Since the resulting residual activities of these genotypes were ≤20%, all new mutations are clearly associated with a significant risk of symptomatic disease as is the prevalent mutation c.985A>G with 5% average residual activity [20] and the mutation c.799G>A with 13% activity [21].

We have attempted to use *in silico* analysis to try and predict the effect of the variants identified on enzyme function. These results were largely inconclusive and inconsistent in many cases with direct measurements of enzyme activities (Table S1).

Therefore, the usage of the *in silico* analyses is not an adequate instrument for the determination of the clinical relevance of novel *ACADM* missense mutations.

In conclusion, our results clearly demonstrate that octanoyl-CoA oxidation in lymphocytes is currently the most efficient and reliable tool for risk assessment of different especially novel *ACADM* genotypes. Genotypes resulting in residual activities ≤20% are clearly disease-causing. Individuals with activities between 20% and 30% would need special supervision and follow-up, whereas activities beyond 30% are not associated with symptomatic disease. We identified mutations that are not associated with a risk of clinical symptomatic MCAD deficiency but must be considered as innocuous variant. Importantly, also these individuals are identified by newborn screening and it is essential to disclose MCAD deficiency in this cohort. In addition, we want to make the assay methods available to health care providers and metabolic laboratories in countries screening for MCAD deficiency in order to correctly decide about the adequate medical treatment only in patients at risk.

## Supporting Information

Table S1 **Literature research and** ***in silico*** **analysis of missense and nonsense mutations of this cohort.** The clinical situation of the patients with the described missense and nonsense mutations was categorized as following: The patient was **affected** if there were any clinical symptoms in the past in patients carrying the mutation in question; the patient was characterized as **carrier**, if no clinical symptoms occurred so far. Mutations were denoted as novel mutation, if they were not described in the literature so far.
(DOC)
