# Supplementary material for: Functional Effects of Different Medium-Chain Acyl-CoA Dehydrogenase Genotypes and Identification of Asymptomatic Variants
Source: PLoS One. 2012 Sep 17;7(9):e45110. doi: 10.1371/journal.pone.0045110 (PMC3444485; doi:10.1371/journal.pone.0045110)
Supplement: Table S1 — Literature research and in silico analysis of missense and nonsense mutations of this cohort. The clinical situation of the patients with the described missense and nonsense mutations was categorized as following: The patient was affected if there were any clinical symptoms in the past in patients carrying the mutation in question; the patient was characterized as carrier, if no clinical symptoms occurred so far. Mutations were denoted as novel mutation, if they were not described in the literature so far. (DOC) [file pone.0045110.s001.doc]

| **Table S1:** **literature research and** *in silico* **analysis of missense and nonsense mutations of this cohort** | | | | | | |
| --- | --- | --- | --- | --- | --- | --- |
| **mutation** | **protein** | **POLYPHEN** | **SNAP** | **Pmut** | **MUpro** | **literature** |
| c.2T>G | p.M1R | probably damaging | non-neutral | PATHOLOGICAL | -0.12270181 | novel mutation |
| c.85C>T | p.R29X | benign | - | - | -0.31631396 | affected |
| c.127G>A | p.E43K | benign | neutral | PATHOLOGICAL | -0.28605691 | carrier |
| c.199T>C | p.Y67H | benign | neutral | NEUTRAL | -1 | carrier |
| c.233T>C | p.I78T | benign | non-neutral | NEUTRAL | -1 | affected |
| c.464T>C | p.M155T | possibly damaging | non-neutral | NEUTRAL | -1 | affected |
| c.533A>C | p.K178T | possibly damaging | non-neutral | PATHOLOGICAL | -0.81020244 | novel mutation |
| c.616C>T | p.R206C | probably damaging | non-neutral | PATHOLOGICAL | -0.99192803 | affected |
| c.698T>C | p.I233T | benign | neutral | NEUTRAL | -1 | affected |
| c.799G>T | p.G267R | possibly damaging | non-neutral | PATHOLOGICAL | 0.74393442 | affected |
| c.977T>C | p.M326T | probably damaging | non-neutral | NEUTRAL | -0.76746561 | affected |
| c.982A>G | p.M328V | benign | neutral | NEUTRAL | -1 | novel mutation |
| c.985A>G | p.K329E | benign | neutral | NEUTRAL | 0.51849322 | affected |
| c.1010A>C | p.Y337S | probably damaging | neutral | PATHOLOGICAL | -0.71322747 | novel mutation |
| c.1033G>T | p.D345Y | probably damaging | non-neutral | NEUTRAL | -0.18060873 | novel mutation |
| c.1225C>T | p.L409F | possibly damaging | non-neutral | NEUTRAL | -0.52935173 | novel mutation |
| c.1229T>G | p.I410S | probably damaging | non-neutral | PATHOLOGICAL | -1 | novel mutation |

1. Derks TG, Reijngoud DJ, Waterham HR, Gerver WJ, van den Berg MP, et al. (2006) The natural history of medium-chain acyl CoA dehydrogenase deficiency in the Netherlands: clinical presentation and outcome. J Pediatr 148: 665-670.

2. McKinney JT, Longo N, Hahn SH, Matern D, Rinaldo P, et al. (2004) Rapid, comprehensive screening of the human medium chain acyl-CoA dehydrogenase gene. Mol Genet Metab 82: 112-120.

3. Zschocke J, Schulze A, Lindner M, Fiesel S, Olgemoller K, et al. (2001) Molecular and functional characterisation of mild MCAD deficiency. Hum Genet 108: 404-408.

4. Andresen BS, Dobrowolski SF, O'Reilly L, Muenzer J, McCandless SE, et al. (2001) Medium-chain acyl-CoA dehydrogenase (MCAD) mutations identified by MS/MS-based prospective screening of newborns differ from those observed in patients with clinical symptoms: identification and characterization of a new, prevalent mutation that results in mild MCAD deficiency. Am J Hum Genet 68: 1408-1418.

5. Yang BZ, Ding JH, Zhou C, Dimachkie MM, Sweetman L, et al. (2000) Identification of a novel mutation in patients with medium-chain acyl-CoA dehydrogenase deficiency. Mol Genet Metab 69: 259-262.

6. Smith EH, Thomas C, McHugh D, Gavrilov D, Raymond K, et al. (2010) Allelic diversity in MCAD deficiency: the biochemical classification of 54 variants identified during 5 years of ACADM sequencing. Mol Genet Metab 100: 241-250.

7. Yokota I, Coates PM, Hale DE, Rinaldo P, Tanaka K (1991) Molecular survey of a prevalent mutation, 985A-to-G transition, and identification of five infrequent mutations in the medium-chain Acyl-CoA dehydrogenase (MCAD) gene in 55 patients with MCAD deficiency. Am J Hum Genet 49: 1280-1291.

8. Andresen BS, Jensen TG, Bross P, Knudsen I, Winter V, et al. (1994) Disease-causing mutations in exon 11 of the medium-chain acyl-CoA dehydrogenase gene. Am J Hum Genet 54: 975-988.

9. Matsubara Y, Narisawa K, Miyabayashi S, Tada K, Coates PM, et al. (1990) Identification of a common mutation in patients with medium-chain acyl-CoA dehydrogenase deficiency. Biochem Biophys Res Commun 171: 498-505.
